# Conversion therapy for hepatocellular carcinoma: paradigm shifts, unresolved controversies, and translational horizons

**DOI:** 10.1016/j.iliver.2026.100271

**Published:** 2026-09-23

**Authors:** Da-Long Yang, Masatoshi Kudo, Tian Yang, Jian-Hong Zhong

**Affiliations:** a Department of Hepatobiliary Surgery, Guangxi Medical University Cancer Hospital, Nanning 530021, China; b Department of Gastroenterology and Hepatology, Kindai University Faculty of Medicine and University Hospital, Sakai 590-0197, Japan; c Department of Hepatobiliary Surgery, Eastern Hepatobiliary Surgery Hospital, Naval Medical University, Shanghai 200438, China; d Key Laboratory of Early Prevention and Treatment for Regional High-Frequency Tumors (Guangxi Medical University), Ministry of Education, Nanning 530021, China; e Guangxi Key Laboratory of Early Prevention and Treatment for Regional High-Frequency Tumors, Nanning 530021, China

**Keywords:** Hepatocellular carcinoma, Conversion therapy, Complete response, Hepatectomy

The synergistic combination of tyrosine kinase inhibitors (TKIs), immune checkpoint inhibitors (ICIs), and transarterial chemoembolization (TACE) represents a therapeutic regimen that substantially improves outcomes in hepatocellular carcinoma (HCC). The primary objective of “*conversion therapy*” is to enable curative resection by downstaging the tumor in patients with initially unresectable HCC. Using this strategy, an inherently incurable HCC can be reclassified as potentially curable, and it can markedly prolong the overall survival (OS) in appropriately selected patients. Increasing retrospective real-world evidence consistently indicates that patients who undergo conversion therapy followed by curative hepatectomy can achieve a 3-year OS rate of approximately 80%, which is comparable to that of patients with initially resectable early-stage HCC.^1^ Despite these encouraging survival outcomes, many critical issues remain unresolved, including the identification of optimal conversion regimens, accurate prediction of treatment-responsive patient subsets, determining the need for surgical resection following radiological or even clinical complete response, and establishment of standardized postoperative adjuvant treatment strategies. This editorial examines consensus guideline recommendations, prospective clinical trial data, and findings from large-scale real-world retrospective cohorts to address core clinical dilemmas faced by frontline hepatobiliary practitioners. The predominant clinical bottlenecks are further delineated, and priority research directions for advancing HCC conversion therapy are outlined.

Major international guidelines substantially differ in their conceptualization of conversion therapy, primarily driven by diverse HCC epidemiological profiles and regional therapeutic approaches. The Chinese guideline formally considers conversion therapy as a standardized management module for unresectable HCC, although the supporting recommendations are generally based on low-certainty evidence because of the scarcity of published large-sample randomized controlled trials (RCTs) and studies using heterogeneous regimen designs.^2^ Western HCC clinical guidelines, including Barcelona Clinic Liver Cancer,^3^ European Association for the Study of the Liver,^4^ National Comprehensive Cancer Network, American Association for the Study of Liver Diseases, and European Society for Medical Oncology do not report the definition of conversion therapy of HCC. Furthermore, insufficient high-level evidence and the lack of definitive RCT data preclude the formulation of formal treatment recommendations.

Over the past several years, numerous small-sample phase II trials have evaluated diverse multimodal regimens for unresectable HCC conversion. To date, only the phase III TALENTOP trial has rigorously investigated surgical resection versus maintenance therapy after atezolizumab plus bevacizumab in patients with unresectable HCC and macrovascular invasion.^5^ With a median randomized follow-up of 18.4 months, the surgical resection strategy significantly prolonged the median time to treatment failure (20.4 vs. 11.8 months) and reduced the risk of disease progression or all-cause mortality by 40%, while also demonstrating a consistent favorable trend in median OS. However, Its primary endpoint was time to treatment failure, not conversion to resection.

Large real-world cohorts constitute the primary evidence base supporting the clinical adoption of conversion therapy, compensating for the scarcity of prospective RCT data. A meta-analysis involving 36 studies and 3497 patients with unresectable HCC revealed an overall pooled conversion rate of 23%, with substantial variability across therapeutic modalities.^6^ Combination regimens integrating TACE with TKIs and ICIs yielded the highest conversion rate of 30%, whereas atezolizumab plus bevacizumab yielded a conversion rate of approximately 4%. Cumulative real-world data indicate that the combination of TACE with lenvatinib and PD-1 inhibitors is a favorable conversion strategy with a manageable safety profile for initially unresectable HCC. The curative hepatectomy rate following TACE monotherapy conversion stood at 23.5%, increasing to 36.4% on combining TACE with TKIs and ICIs.^7^

Treatment selection requires risk stratification based on the etiology of unresectability, underlying cirrhosis severity, baseline hepatic functional reserve, and viral hepatitis status to avoid excessive hepatic toxicities that can potentially delay definitive surgery. A systematic review of recent RCTs indicated that monotherapy with TKIs or ICIs or dual anti-angiogenic-ICI regimens yield objective response rates (ORR) of <30%, with median progression-free survival ranging from 2.9 to 9.2 months and a consistent median OS of <24 months. These outcomes were obtained from prospective trial populations, whereas efficacy endpoints for triple therapy combining TACE, TKIs, and ICIs are derived from retrospective real-world cohorts. Cross-study comparisons are limited by heterogeneity in patient ethnicity, tumor stage, and study design. In a large multicenter retrospective study, triple therapy achieved an ORR of >50% and a median progression-free survival of 15.9 months; median OS was not reached, and the 3-year OS rate was 56.2%.^7^ Survival benefits of conversion therapy substantially vary by disease stage. A head-to-head large-sample study comparing TACE triple therapy with TACE monotherapy reported robust survival improvements in patients with intermediate and advanced HCC, whereas no statistically significant intergroup differences in efficacy were observed in patients with early-stage unresectable HCC.^7^ Although triple-modality regimens confer consistent survival advantages in most intermediate-/advanced-stage cases, refined TACE monotherapy may be sufficient to improve long-term outcomes in selected patients with early-stage unresectable HCC.

Whether curative hepatectomy improves long-term prognosis in cases rendered resectable following conversion therapy remains one of the most contentious unresolved clinical questions. Disease progression remains common even among patients achieving radiological or clinical complete response without hepatectomy. Nevertheless, postoperative tumor recurrence is also observed in patients who undergo resection following conversion therapy. Comparative cohort analyses revealed comparable rates of disease progression in nonsurgical patients and tumor recurrence in patients with resectable disease following partial or complete conversion response. However, the hepatectomy group achieved markedly superior long-term OS.^1^ Hepatectomy confers a consistent survival benefit in patients with partial or complete response to conversion therapy. However, for individuals achieving radiological or clinical complete response with excellent long-term survival, the need to endure the risks and trauma of surgical resection remains under debate. Notably, radiological complete response does not equate to pathological complete response (pCR). Among patients with radiological complete response after conversion therapy, only 67.1% achieved pCR upon hepatectomy; this proportion increased to 78.5% in those with complete response. Viable residual tumor cells were identified in 21.5%–32.9% of surgical specimens, alongside occult micrometastases and peritumoral satellite lesions undetectable by cross-sectional imaging, pathological features that increase the risk of subsequent disease progression. Hepatectomy is associated with inherent procedural risks, including perioperative complications and mortality. Accordingly, heightened clinical vigilance is mandatory for patients with advanced age, severe cirrhosis, multiple comorbidities, or anticipated extensive resection of normal hepatic parenchyma. Locoregional alternatives, such as definitive radiotherapy, represent reasonable alternative curative-intent strategies for patients at high surgical risk.

Patients undergoing curative hepatectomy following conversion therapy remain at substantial risk of postoperative recurrence, primarily due to preoperative microvascular invasion and occult residual tumor cells. Two pivotal global multicenter phase III RCTs (IMbrave050^8^ and KEYNOTE-937) failed to demonstrate that adjuvant atezolizumab–bevacizumab or single-agent pembrolizumab therapy reduces HCC recurrence after curative resection. To date, no standardized adjuvant regimen has been established for patients undergoing hepatectomy after conversion therapy, even among those with high-risk pathological features predictive of recurrence. A recent retrospective analysis revealed comparable long-term survival between active surveillance and adjuvant ICI therapy among patients who achieved pCR following conversion therapy.^9^ In routine clinical practice, antiviral suppression and structured surveillance constitute the dominant postoperative management paradigm. Overly intensive adjuvant regimens may further impair limited hepatic functional reserve, potentially restricting the use of subsequent salvage antitumor therapies upon recurrence.

Several key issues remain unresolved. First, validated predictive biomarkers are lacking. Second, no head-to-head RCT compares conversion therapy plus resection with continued palliative systemic therapy. Third, the optimal timing between conversion completion and hepatectomy is undefined. Fourth, conversion rates widely vary across centers. Fifth, the optimal surgical criteria following conversion therapy remain undefined, with ongoing debate regarding the need for anatomic resection and >1 cm wide margins as well as the safety of capsule-preserving resection. Tumor necrosis blurs tumor boundaries, thereby limiting traditional anatomic-resection principles. Retrospective studies prioritize microscopic R0 status over fixed margin width, with 0.1 cm margins yielding comparable survival outcomes. However, there is limited evidence supporting capsule-preserving resection, highlighting the need for prospective data. Four priority research directions are proposed: (1) large international multicenter RCTs to validate the survival benefit of conversion therapy followed by resection; (2) multi-omics predictive biomarkers to enable upfront patient stratification; (3) risk-stratified perioperative adjuvant protocols based on postoperative pathological response; and (4) harmonized international definitions distinguishing conversion therapy from neoadjuvant therapy to standardize trial design. Novel multimodal regimens may further help improve response and conversion rates.

Conversion therapy has markedly redefined the therapeutic paradigm for unresectable HCC, delivering curative hepatectomy opportunities to millions of patients previously limited to palliative systemic therapy. Accumulating evidence from guideline consensus, prospective trial readouts, and large real-world cohorts supports its clinical utility. Nevertheless, important uncertainties remain regarding optimal regimen selection, individualized patient eligibility, the need for hepatectomy following treatment response, and standardized postoperative surveillance and adjuvant strategies, highlighting the need for high-quality prospective clinical research. The persistent cross-regional conceptual divergence between Chinese and Western guidelines further highlights the urgent need for unified international clinical frameworks governing preoperative HCC treatment. Guided by multidisciplinary team-driven precise patient stratification and supported by emerging predictive biomarkers, standardized multimodal conversion therapy should further improve durable long-term survival among patients with unresectable HCC in the era of targeted and immune therapies.

## CRediT authorship contribution statement

**Da-Long Yang:** Writing – original draft, Data curation, Conceptualization. **Masatoshi Kudo:** Writing – original draft, Conceptualization. **Tian Yang:** Conceptualization, Data curation, Writing – review & editing. **Jian-Hong Zhong:** Writing – review & editing, Formal analysis, Conceptualization.

## Informed consent

Not applicable.

## Ethics statement

Not applicable.

## Organ donation

Not applicable.

## Animal treatment

Not applicable.

## Data availability statement

Not applicable as no data sets were generated or analyzed.

## Declaration of generative AI and AI-assisted technologies in the writing process

Not applicable.

## Funding

None.

## **Declarationofcompetinginterest**

The author Tian Yang is Executive Editor-in-Chief and Jian-Hong Zhong is a member of the editorial board for this journal and they were not involved in the editorial review or the decision to publish this article.
